# Impact of bisphosphonates on the proliferation and gene expression of human fibroblasts

**DOI:** 10.7150/ijms.36994

**Published:** 2019-10-21

**Authors:** Francisco Javier Manzano-Moreno, Rebeca Illescas-Montes, Lucia Melguizo-Rodriguez, Victor J. Costela-Ruiz, Olga García-Martínez, Concepción Ruiz, Javier Ramos-Torrecillas

**Affiliations:** 1 Biomedical Group (BIO277), Department of Stomatology, School of Dentistry, University of Granada, Spain.; 2 Biomedical Group (BIO277), Department of Nursing, Faculty of Health Sciences. University of Granada, Spain.; 3 Instituto Investigación Biosanitaria, ibs.Granada, Spain.; 4 Institute of Neuroscience, Parque Tecnológico Ciencias de la Salud, Armilla (Granada), University of Granada, Spain.

**Keywords:** bisphosphonates, osteonecrosis, jaw, fibroblast, gene expression

## Abstract

The aim of this study was to elucidate the role of fibroblasts in bisphosphonate-related osteonecrosis of the jaw (BRONJ), evaluating the effect of zoledronate, alendronate, and ibandronate on the proliferation of fibroblasts and on their expression of genes essential for fibroblast physiology. Human CCD-1064Sk epithelial fibroblast cells were incubated in culture medium with 10^-5^, 10^-7^, or 10^-9^ M zoledronate, alendronate, or ibandronate. The proliferative capacity of fibroblasts was determined by spectrophotometry (MTT) at 24 of culture. Real-time polymerase chain reaction (RT-PCR) was used to study the effects of BPs at a dose of 10^-9^ M on the expression of FGF, CTGF, TGF-β1, TGFβR1, TGFβR2, TGFβR3, DDR2, α-actin, fibronectin, decorin, and elastin. Fibroblasts proliferation was significantly increased at the lowest dose (10^-9^M) of each BP but was not affected at the higher doses (10^-5^ and 10^-7^M). The proliferation increase may be related to the rise in TGF-β1 and TGFβR1 expression detected after the treatment of cells with 10^-9^M of zoledronate, alendronate, or ibandronate. However, the expression of CTGF, DDR2, α-actin, fibronectin, and decorin decreased *versus* controls. The results of this *in vitro* study indicate that a very low BP dose (10^-9^ M) can significantly affect the physiology of fibroblasts, increasing their proliferative capacity and modulating the expression of multiple genes involved in their growth and differentiation.

## Introduction

Bisphosphonates (BPs) are synthetic analogs of pyrophosphate in which the carbon replaces the oxygen linking the phosphates. They are commonly used for the treatment of some bone disorders like osteoporosis, Paget´s disease, multiple myeloma, and malignant hypercalcemia.^1^ There are two types of BP: nitrogen-containing BPs and non-nitrogen-containing BPs.^2^ The effectiveness of these drugs has been demonstrated by several studies , but they have also been associated with the development of BP-related osteonecrosis of the jaw (BRONJ).^3^

A reduction in bone turnover and consequent accumulation of microfractures, an anti-angiogenic effects of BPs, and an alteration on the viability of fibroblasts and oral keratinocytes have been associated with the development of BRONJ.^4^ Previous studies of our research group demonstrated that high doses of BPs have toxic effects on osteoblasts 5 and that low doses reduce their differentiation capacity.^6^ However, although BPs are known to suppress bone turnover, the mechanism by which this translates into ulceration of the overlying mucosa remains unclear. BPs are known to affect mucosal tissues at high concentrations, but the clinical relevance of this effect is unknown.^7^

Fibroblasts are responsible for forming and maintaining soft connective tissue and constitute the main source of collagen for the extracellular matrix (ECM). They are in a quiescent state in healthy conjunctive tissue, being metabolically active but unable to proliferate, although they slowly synthetize, degrade, and organize the ECM to maintain the tissue structure.^8^ However, tissue injuries induce major changes in cell signaling that translate into cell activation, stimulating the formation of granulation tissue with a high component of cells (fibroblasts, macrophages, myofibroblasts, neovasculature), which contributes to the creation of new mature connective tissue and favors tissue regeneration.^8^

The objective of this study was to determine the effects of treatment with three nitrogen-containing BPs (zoledronate, alendronate, and ibandronate) on the proliferation of fibroblasts and on their expression of genes essential for fibroblast physiology.

## Material and Methods

### Cell cultures

The human CCD-1064Sk epithelial fibroblast cell line was purchased from American Type Cultures Collection (ATCC, Manassas, VA, USA) (ATCC: CRL-2076) and maintained in Dulbecco's Modified Eagle Medium (DMEM; Invitrogen Gibco Cell Culture Products, Carlsbad, CA) with 100 IU/mL penicillin (Lab Roger SA, Barcelona, Spain), 50 μg/mL gentamicin (Braum Medical SA, Jaen, Spain), 2.5 μg/mL amphotericin B (Sigma -Aldrich Co. Chem. Comp., St. Louis, Mo, USA), 1 % glutamine (Sigma -Aldrich Co), and 2 % HEPES (Sigma -Aldrich Co) supplemented with 10 % fetal bovine serum (FBS) (Gibco, Paisley, UK). Cultures were kept at 37 ºC in humidified atmosphere of 95 % air and 5 % CO_2_. Cells were detached from the culture flask with a solution of 0.05 % trypsin (Sigma-Aldrich Co) and 0.02 % ethylene diamine tetra-acetic acid (EDTA) (Sigma -Aldrich Co) and were then washed and suspended in complete culture medium with 10 % FBS. The study was approved by the Ethics Committee of the University of Granada.

### Treatments

The human CCD-1064Sk epithelial fibroblast cell line was treated for 24 h with zoledronate (Sigma-Aldrich, St. Louis, MO), alendronate (Sigma), or ibandronate (Sigma-Aldrich) at doses of 10^-5^, 10^-7^, or 10^-9^ M, which are within the therapeutic dose range.^10^

### Cell proliferation assay

Fibroblasts were seeded at 1 x 10^4^ cells/mL per well into a 24-well plate (Falcon, Becton Dickinson Labware, NJ) and cultured at 37 ºC in a humidified atmosphere of 95% air and 5% CO_2_ for 24 h. Next, the medium was replaced with DMEM containing zoledronate, alendronate, or ibandronate at a dose of 10^-5^, 10^-7^, or 10^-9^ M. After 24 h of culture, cell proliferation was measured by MTT assay, as described by Manzano-Moreno *et al.* (2013).^11^ Results were expressed with respect the control. At least three experiments were conducted for each treatment, using the mean value for data analyses.

### RNA extraction and cDNA synthesis (reverse transcription)

The method described by Manzano-Moreno *et al.* (2018)^12^ was used to extract the mRNA of cells treated with 10^-9^ M of zoledronate, alendronate and ibandronate and of control cells cultured under the same conditions. All assays were run in triplicate. Subsequently, an equal amount of RNA (1 μg total RNA in 40 μL total volume) was reverse-transcribed to cDNA and amplified by PCR using the iScript™ cDNA Synthesis Kit (Bio-Rad laboratories, Hercules, CA) in accordance with the manufacturer`s instructions.

### Real-time polymerase chain reaction (q-RT-PCR)

The mRNA of fibroblast growth factor (FGF), connective tissue growth factor (CTGF), transforming growth factor β1 (TGF-β1), transforming growth factorβ-receptors (TGFβR1, TGFβR2, and TGFβR3), discoidin domain receptor 2 (DDR2), α-actin, fibronectin, decorin, and elastin were detected with primers designed using the NCBI-nucleotide library and Primer3-design (Table 1). All primers were designed with NCBI Blast software. Ubiquitin C (UBC), peptidylprolyl isomerase A (PPIA), and ribosomal protein S13 (RPS13) were used as stable housekeeping genes to normalize the final results.^13^

Quantitative RT-PCR (q-RT-PCR) was performed using the SsoFast™ EvaGreen® Supermix Kit (Bio-Rad laboratories) in accordance with the manufacturer`s protocol. Standard curves were constructed for each target gene by plotting Ct values versus log cDNA dilution. After each real-time RT-PCR, a melting profile was created and agarose gel electrophoresis of each sample was carried out in order to rule out non-specific PCR products and primer dimers. For the relative quantification of gene expression the comparative Ct method was applied. The mRNA concentration of each gene was expressed in ng of mRNA per average ng of housekeeping mRNAs.

### Statistical analysis

For the data analyses SPSS 22.0 (IBM, Chicago, IL) was used. Mean values (±SD) were calculated for each variable. ANOVA was performed to examine the effects on proliferation and mRNA levels. When a significant interaction was identified, the Bonferroni correction was applied for planned pair-wise comparisons. At least three experiments were conducted for all assays. P ≤ 0.05 was considered statistically significant in all tests.

## Results

### Cell proliferation assay

**Figure 1:**
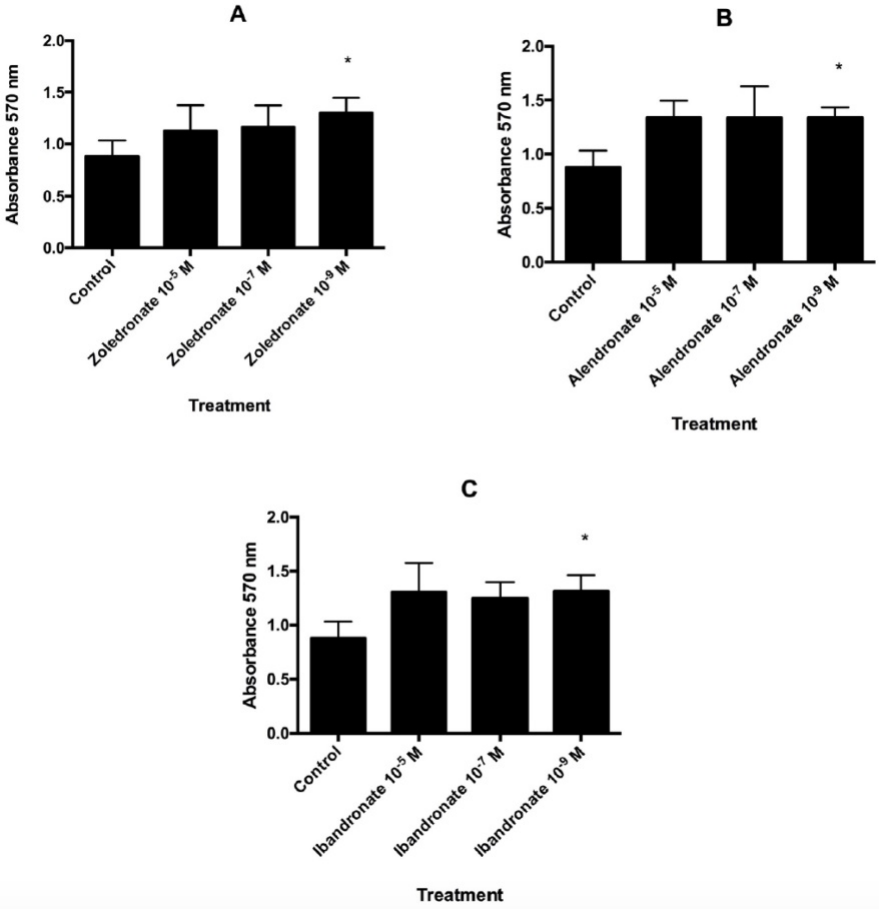
Effect of A) zoledronate; B) alendronate; C) ibandronate at different doses (10^-5^ M, 10^-7^ M, 10^-9^ M) on fibroblast proliferation after 24 h of incubation. Data are expressed as means + SD. *p ≤ 0.05

Each amino-BP under study stimulated fibroblast proliferation capacity as a function of the dose (Fig. 1). In comparison to controls, a significant increase in proliferation was observed in BP-treated cells at the lowest dose assayed (10^-9^ M): zoledronate (p=0.004), alendronate (p=0.007), and ibandronate (p=0.023).

### Effect of bisphosphonates on the expression of genes encoding different growth factors (TGF-β1, TGF-β1 receptors, FGF, and CTGF)

**Figure 2:**
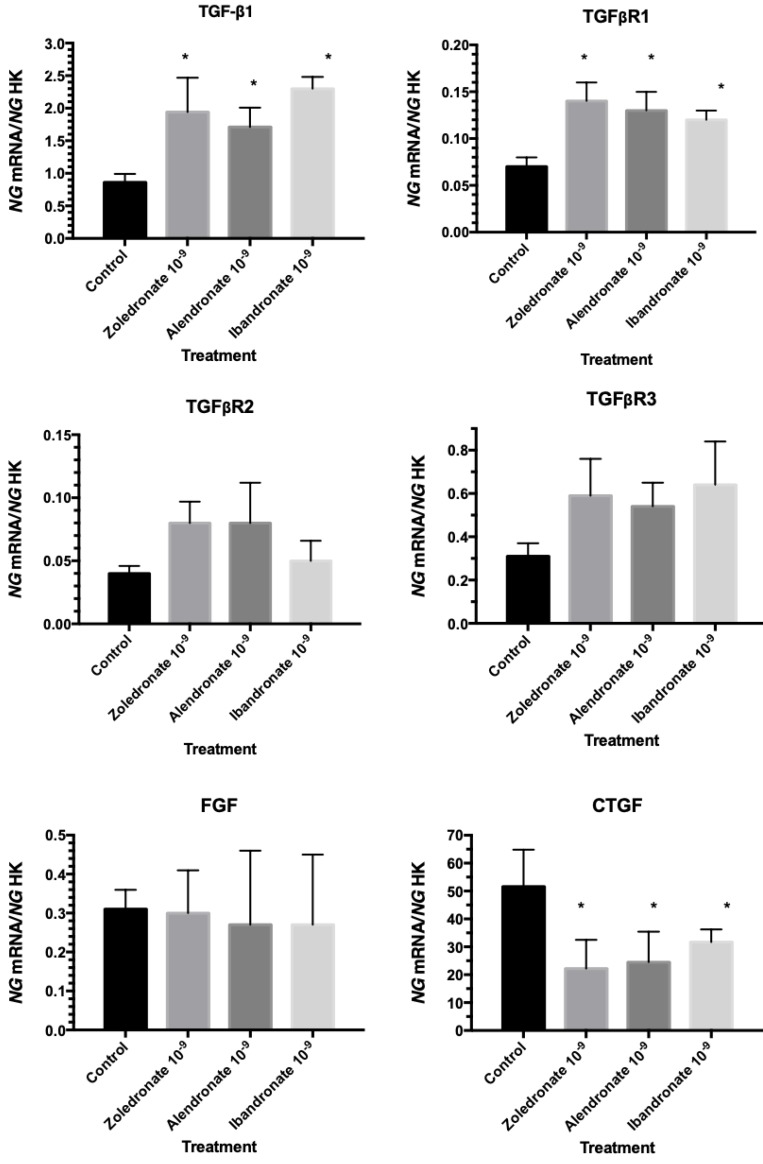
Expression of fibroblast genes (TGF-β1, TGFβR1, TGFβR2, TGFβR3, FGF, and CTGF) treated with zoledronate, alendronate, or ibandronate at a dose of 10^-9^M. Data are expressed as ng of mRNA per average ng of housekeeping mRNAs ± SD. *p ≤ 0.05

Quantitative RT-PCR (q-RT-PCR) analysis was used to evaluate the expression of growth factors involved in fibroblast physiology. As depicted in Figure 2, cells treated with the BPs at a dose of 10^-9^ M showed increased TGF-β1 and TGFβR1 gene expression, with no significant changes in FGF, TGFβR2, or TGFβR3 gene expression, and a significant reduction in CTGF gene expression.

### Effect of bisphosphonates on the expression of genes encoding α-actin, fibronectin, decorin, elastin, and DDR2

**Figure 3:**
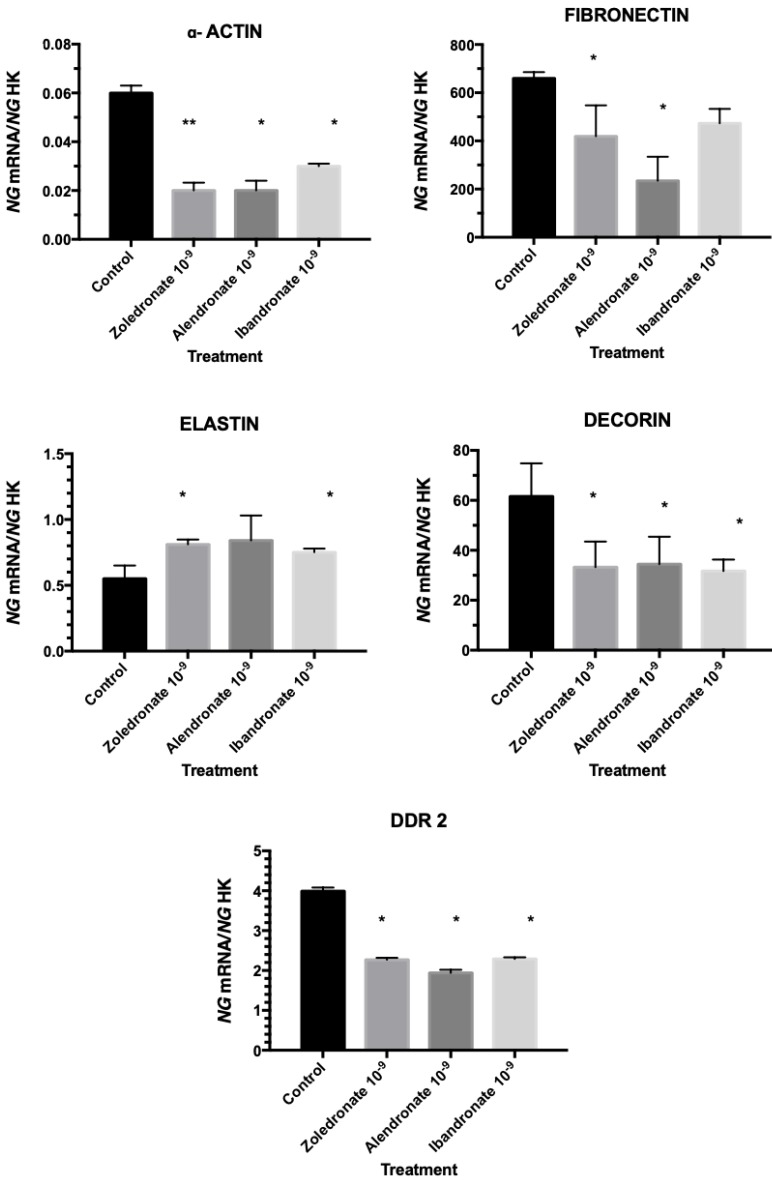
Expression of fibroblasts genes (α-actin, fibronectin, decorin, elastin, and DDR2) treated with zoledronate, alendronate, or ibandronate at dose of 10^-9^M. Data are expressed as ng of mRNA per average ng of housekeeping mRNAs ± SD. *p ≤ 0.05; *p ≤ 0.001

Figure 3 depicts the q-RT-PCR results obtained for the expression of genes encoding α-actin, fibronectin, decorin, elastin, and DDR2. In comparison to controls, all three treatments significantly reduced the expression of all of these genes with the exception of the gene for elastin, whose expression was significantly increased.

## Discussion

The present study demonstrates that *in vitro* treatment with nitrogen-containing BPs at a dose of 10^-9^ M increases fibroblast proliferation and modulates the expression of the human fibroblast markers, TGF-β1, TGFβR1, CTG, α-actin, fibronectin, decorin, elastin, and DDR2. The proteins encoding these markers play a major role in wound healing by stimulating fibroblast proliferation, migration, and/or maturation.^14^ The proliferation of fibroblasts plays a key role in maintaining soft tissue integrity and regeneration and was increased by treatment with zoledronate, alendronate, or ibandronate, although only at the lowest dose assayed (10^-9^ M); no significant changes were detected at doses of 10^-5^ or 10^-7^ M. The three doses assayed are within the therapeutic dose range.^10^

McLeod *et al.* 2014 7 reported that alendronate suppressed cell proliferation at 100 μM in human fibroblasts, a much higher dose than those assayed in our study. Martins *et al.* 2015 15 then observed that alendronate can inhibit human fibroblast proliferation at doses as low as 10 μM. The response of fibroblasts to BPs in our study is similar to that observed in human osteoblasts, whose proliferative capacity was stimulated at very low BP doses but not at higher doses, with the observation of toxic effects.^5^,^6^ Song *et al.* 2018 16 recently reported that the cytotoxic effect of BPs on fibroblastic cells depends on their dose and concentration. Although a dose of 10^-9^ M is within the therapeutic range of BPs, there is no knowledge of the actual concentration reached in soft tissue when the drug is released from bone hydroxyapatite crystals, where it accumulates during long-term BP treatments.^17^

A complex interplay of different cell types (osteogenic cells, oral keratinocytes, fibroblasts, and endothelial cells) is needed for a correct wound healing in the oral cavity.^18^ BRONJ most commonly appears after injury to the oral tissues (e.g., after dental extraction), and its pathophysiology may arise from an effect on these multiple cell types.

BPs may compromise the function of fibroblasts and vessel cells, impairing oral mucosa re-epithelialization and nutrition supply.^19^ Compromised cell function and viability are considered to contribute to BRONJ onset alongside a fragile and vulnerable oral environment due to thin mucosal coverage, microflora, chewing, and frequent dental procedures.^20^

TGF-β1 exerts multiple functions, including the stimulation of fibroblast proliferation, migration, and adhesion and the promotion of ECM element production.^19^ TGF-β1 also favors the maturation of fibroblasts, inducing their differentiation to myofibroblasts, which are responsible for contractions and for synthetizing ECM elements.^21^ In the present study, TGF-β1 expression was significantly increased after treatment with a low dose of BP (10^-9^ M), which would explain the increased proliferative capacity observed at this dose alongside the treatment-induced increase in expression of one of the TGF-β1 receptors (TGFβR1). However, no changes were found in the expression of FGFs, the main growth factors for this cell population.^22^ The BP treatment produced a decrease in the expression of CTGF, a multifactorial growth factor that participates in ECM regulation and synthesis, endothelial cell migration, angiogenesis, and fibroblast proliferation and differentiation, among other processes.^23^ A decrease in CTGF expression may affect the regeneration of both hard and soft tissues, whose alteration may be the main cause of BRONJ development.

Markers α-actin, fibronectin, decorin, and DDR-2 are strongly related to fibroblast differentiation/ maturation and are therefore involved in tissue repair.^24^ DDR2 collagen receptors regulate fibroblast proliferation and migration and ECM synthesis, which are crucial in wound-healing. There is also a close relationship between DDR2 and MMP-2, predominant proteases in the ECM and responsible for wound remodeling. Thus, a decrease in DDR2 was reported to reduce migration and MMP-2 expression in fibroblasts.^23^ In the present study, BP treatment decreased the expression of DDR-2 in human fibroblasts, which may imply inhibition of their migration and MMP-2 expression.

Treatment with the studied BPs at dose of 10^-9^M was found to increase the expression of elastin, and an increase in elastin fibers is known to facilitate fibroblast proliferation.^25^ It also reduced the expression of decorin, which may compromise tissue repair because the functions of proteoglycan decorin include the regulation of collagen fiber production and organization of the ECM alongside the enhancement of growth factor bioavailability in this matrix.^26^

Low-dose BP treatment significantly reduced the expression of myofibroblast markers α-actin and fibronectin. Myofibroblasts present in granulation tissue possess intermediate characteristics between fibroblasts and smooth muscle cells and play a major role in the inflammation, repair, and remodeling of tissues. They differentiate from fibroblasts and are characterized by the expression of α-actin.^27^ The significant reduction in these markers may alter the differentiation of fibroblasts and therefore their role in wound regeneration.

Studies of osteoblasts by our group 5,6,28 demonstrated an increase in their proliferation at low concentrations of BPs, similar to the present findings in fibroblasts, with a decrease in their differentiation capacity and reductions in alkaline phosphatase activity, mineralization, and the expression of genes related to cell differentiation. Likewise, the present study found that a low dose of BPs reduces the expression of certain genes related to fibroblast differentiation. These findings may be directly related to the development of BRONJ through loss of the capacity for adequate oral soft tissue repair after surgical aggression such as tooth extraction.

The present results indicate that treatment with low BP doses increases the proliferation of fibroblasts but reduces the gene expression of markers involved in their migration and differentiation of this population. Our findings are in concordance whit the study of Zafar *et al.* 29 that showed an increase in fibroblasts growth treated with low doses of bisphosphonates at short term, meanwhile long-term treatment exhibited an adverse effect. These results are related with the changes we observed in the gene expression study, at 24 h of treatment.

Thereby altering wound healing, which may contribute to BRONJ development in the oral cavity. BRONJ is a multifactorial entity and various proposals have been made to explain its onset, including: a decrease in bone turnover and subsequent accumulation of microfractures; a toxic effect on osteoblasts 5; an adverse effect on osteoclasts 30; an anti-angiogenic effect producing avascular necrosis; and a reduction in the viability of fibroblasts and oral keratinocytes.^31^ However, further studies are required to fully elucidate the effects of long-term BP consumption on the role of fibroblasts in BRONJ.

In conclusion, the administration of BPs at low therapeutic doses increases the proliferative capacity of fibroblasts but reduces the expression of genes essential for their growth and differentiation. These changes may impair the functional capacity of these cells at soft tissue level and thereby contribute to BRONJ development, alongside other factors.

## Figures and Tables

**Table 1:** Primer sequences for the amplification of cDNA by real-time PCR

Gene	Sense Primer	Antisense Primer	Amplicon (bp)
FGF	5´-CCCATATTCCCTGCACTTTG-3´	5´-ACCTTGACCTCTCAGCCTCA-3´	195
CTGF	5´-CCTGGTCCAGACCACAGAGT-3´	5´-TGGAGATTTTGGGAGTACGG-3´	194
TGF-β1	5´-TGAACCGGCCTTTCCTGCTTCTCATG-3´	5´-GCGGAAGTCAATGTACAGCTGCCGC-3´	152
TGFβR1	5´-ACTGGCAGCTGTCATTGCTGGACCAG-3´	5´-CTGAGCCAGAACCTGACGTTGTCATATCA-3´	201
TGFβR2	5´-GGCTCAACCACCAGGGCATCCAGATGCT-3´	5´-CTCCCCGAGAGCCTGTCCAGATGCT-3´	139
TGFβR3	5´-ACCGTGATGGGCATTGCGTTTCCA-3´	5´-GTGCTCTGCGTGCTGCCGATGCTGT-3´	173
DDR2	5´-GAACCCAAACATCATCCATC-3´	5´-CTTCATGCCAGAGGCAATTT-3´	199
α-actin	5´-TCCTGCTCCTCTCTGTCTCAT-3´	5´-AGTCAGAGCTTTGGCTAGGAA-3´	96
fibronectin	5´-GCCATGACAATGGTGTGAAC-3´	5´-GCAAATGGCACCGAGATATT-3´	200
decorin	5´-GGGCTGGCAGAGCATAAGTA-3´	5´-CAGAGCGCACGTAGACACAT-3´	196
elastin	5´-GGTGTAGGTGGAGCTTTTGC-3´	5´-CTGTTGGGTAACCAGCCTTG-3´	199
